# Functional and Structural Alterations in Pediatric Multiple Sclerosis: A Systematic Review and a Preliminary Activation Likelihood Estimation Functional Magnetic Resonance Imaging Meta-Analysis

**DOI:** 10.3390/pediatric17030057

**Published:** 2025-05-13

**Authors:** Nicoletta Cera, Joana Pinto, Ricardo Faustino

**Affiliations:** 1 Faculty of Psychology and Education Sciences, University of Porto, 4200-135 Porto, Portugal; up201707095@up.pt; 2 Cross I&D Lisbon Research Center, Escola Superior de Saúde da Cruz Vermelha Portuguesa, 1300-125 Lisbon, Portugal; 3 Faculty of Medicine, University of Porto, 4200-319 Porto, Portugal; 4 Institute of Biophysics and Biomedical Engineering, Faculty of Science, University of Lisbon, 1749-016 Lisbon, Portugal

**Keywords:** pediatric multiple sclerosis, functional MRI, structural brain alterations, activation likelihood estimation (ALE), cognitive impairment, brain connectivity, neurodevelopment, thalamic atrophy, cerebellar lesions, white matter abnormalities

## Abstract

Background/Objectives: Pediatric multiple sclerosis (MS) is a rare and complex neuroinflammatory disease characterized by demyelination and neurological dysfunction in individuals under 18 years of age. This systematic review and activation likelihood estimation (ALE) meta-analysis aimed to synthesize the existing literature on functional and structural brain alterations in pediatric MS patients. Methods: Following the PRISMA guidelines, we analyzed 21 studies involving 917 pediatric MS patients and 320 healthy controls, assessing brain structure and function using MRI and fMRI techniques. Results: The results reveal consistent alterations in brain regions critical for cognitive and motor functions, including reduced brain volume, increased lesion load, and disrupted functional connectivity, particularly in the thalamus, cerebellum, and hippocampus. The ALE meta-analysis identified significant activation clusters in the dorsal anterior cingulate cortex, angular gyrus, and superior parietal lobes, regions associated with cognition, attention, and working memory. Conclusions: These findings suggest that pediatric MS uniquely affects brain development, contributing to cognitive impairments that differ from those observed in adult MS. Our study underscores the importance of early diagnosis and tailored therapeutic interventions to mitigate neurodevelopmental disruptions and improve long-term outcomes in pediatric MS patients.

## 1. Introduction

Pediatric multiple sclerosis (MS) is a complex and challenging neuroinflammatory disorder characterized by demyelination and subsequent neurological dysfunction in individuals under the age of 18. Pediatric MS is a rare disease with an incidence of 0.13–0.6 cases per 100,000 children per year [1]. Additionally, 2–10% of individuals diagnosed with MS experience their first symptoms during childhood [2]. According to a recent meta-analysis [3], most epidemiological studies reported a higher prevalence of pediatric MS among high-income countries. Most of these studies were carried out in European and Middle Eastern countries [3].

Preadolescent patients presented with multifocal symptoms, whereas adolescent patients showed single-focal symptoms that were more similar to those observed in adults. Among the four subtypes of MS, 95–98% of pediatric patients are classified as relapsing–remitting, and less than 3% of patients are primarily progressive [4]. During the last few years, magnetic resonance imaging (MRI) has played a prominent role in the diagnosis and prediction of the clinical course in adults with MS. In adult patients, at the first attack, the spinal cord and asymptomatic infratentorial lesions are associated with the development of clinical disability as assessed by the EDSS [5]. This type of information is still lacking in the MS pediatric population.

While recent studies have made significant strides in understanding clinical predictors and treatment responses in pediatric MS patients [6,7], a crucial gap remains in comprehensively examining the functional and structural brain alterations specific to this unique patient population. However, only a few longitudinal studies have been carried out to disentangle the clinical course and outcomes in patients who developed MS before the age of 18 years [8,9].

Functional magnetic resonance imaging (fMRI) is a noninvasive technique that is able to investigate the cerebral underpinnings of different cognitive and emotional functions by collecting and studying blood oxygen level-dependent (BOLD) signals [10]. fMRI allows the depiction of both task-related and spontaneous brain activity. According to a recently published review [11], BOLD changes in MS adult patients have been widely studied using motor and visual tasks, which highlighted a complex set of cortical and subcortical brain regions. In adult MS patients, 71 task-based and 22 resting-state fMRI studies were identified in 2018 [12]. Interestingly, most studies have focused on working memory and motor functions, with limited attention given to decision-making. Among the task-based fMRI studies, MS patients with cognitive impairment presented decreased activation of the hippocampal network and no additional activations in the premotor or prefrontal cortex. Resting-state studies, which use different analytical techniques, revealed alterations in the crosstalk among the principal resting-state networks in MS. The default mode network (DMN), one of the principal resting-state networks, was altered in patients with a progressive form of MS. These alterations were more pronounced in patients with cognitive impairment.

Despite these results in the adult population, less is known about functional brain alterations in pediatric patients with MS.

This review seeks to address this gap through a systematic review and activation likelihood estimation (ALE) meta-analysis. Our primary focus is synthesizing literature to provide a comprehensive overview of the functional and structural brain changes observed in pediatric MS. By incorporating findings from recent studies, we aim to offer a nuanced understanding of the neural underpinnings of pediatric MS.

The structural and functional alterations in the developing brain present unique challenges and opportunities for intervention in pediatric MS patients. Our systematic review critically examines the existing evidence and identifies key trends, inconsistencies, and gaps in the literature. Furthermore, the ALE meta-analysis provides a quantitative synthesis of neuroimaging data, offering insights into the spatial convergence of reported brain alterations in pediatric MS patients.

As we embark on this exploration of the pediatric MS neuroimaging landscape, our goal is to contribute to a deeper understanding of the intricate interplay between clinical manifestations, brain structure, and function. By elucidating these relationships, we hope to pave the way for targeted interventions and personalized treatment strategies that address the specific needs of children and adolescents grappling with this challenging neurological condition.

## 2. Materials and Methods

**Figure 1:**
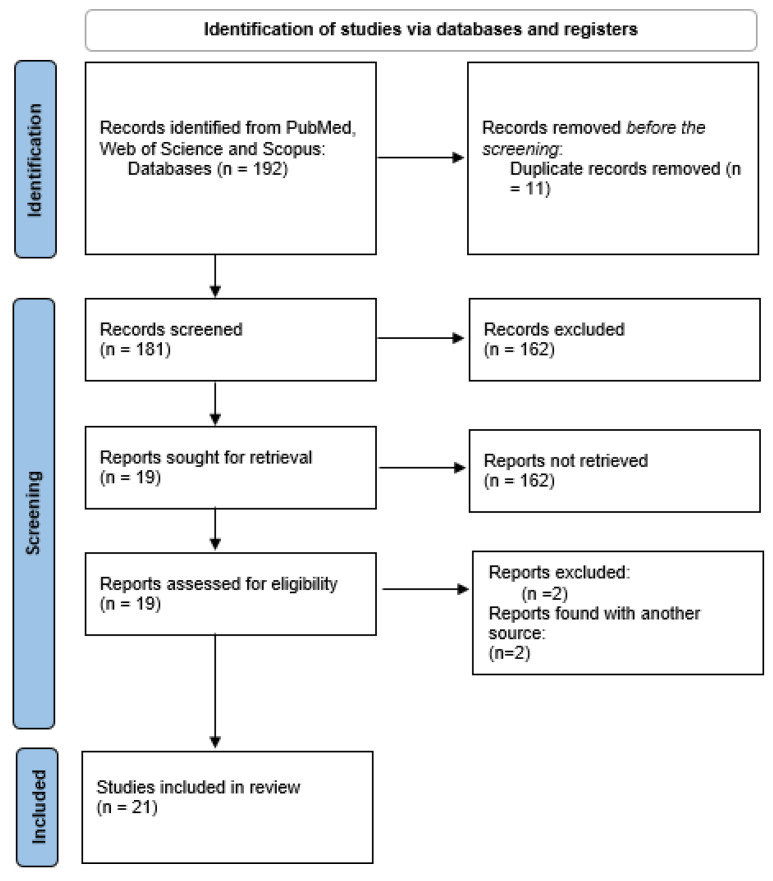
Flowchart of the selection process. *From:* Page MJ, McKenzie JE, Bossuyt PM, Boutron I, Hoffmann TC, Mulrow CD, et al. The PRISMA 2020 statement: an updated guideline for reporting systematic reviews. BMJ 2021;372:n71. doi: 10.1136/bmj.n71 [14]. For more information, visit http://www.prisma-statement.org/ (accessed on 12 March 2024).

The present review followed the Preferred Reporting Items for Systematic Reviews and Meta-Analyses (PRISMA) guidelines and the PICO research question strategy protocol (the PROSPERO registration No. is 1023314) [13]. The flowchart in Figure 1 depicts the steps of the selection process [14].

Our research question was specifically oriented toward functional and structural neural alterations (O) in MS pediatric patients (P) compared with healthy age-matched controls (C), with different methodological approaches. The present systematic review and meta-analysis were limited to MRI rest or task-based fMRI studies (I).

We defined the search terms on the basis of the PICO question mentioned above, combined with the Boolean operators “AND” and “OR”, according to the methods used in other previously published systematic reviews [15,16].

We conducted a computer-based literature search following the PICO approach as follows: “pediatrics” [All Fields] OR “pediatrics” [MeSH Terms] OR “pediatrics” [All Fields] OR “pediatric” [All Fields] OR “pediatric” [All Fields]) AND (“multiple sclerosis” [MeSH Terms] OR (“multiple” [All Fields] AND “sclerosis” [All Fields]) OR “multiple sclerosis” [All Fields]) AND (“magnetic resonance imaging” [MeSH Terms] OR (“magnetic” [All Fields] AND “resonance” [All Fields] AND “Imaging” [All Fields]) OR “magnetic resonance imaging” [All Fields] OR “fmri” [All Fields]) AND (“magnetic resonance imaging” [MeSH Terms] OR (“magnetic” [All Fields] AND “resonance” [All Fields] AND “imaging” [All Fields]) OR “magnetic resonance imaging” [All Fields] OR “mri” [All Fields]) AND (“brain” [MeSH Terms] OR “brain” [All Fields] OR “brains” [All Fields] OR “brain s” [All Fields]) AND (“age of onset” [MeSH Terms] OR (“age” [All Fields] AND “onset” [All Fields]) OR “age of onset” [All Fields] OR “onset” [All Fields] OR “onsets” [All Fields] OR “onsetting” [All Fields]). The literature search was conducted to retrieve all the published articles in English from three principal databases: PubMed (MEDLINE), Scopus, and Web of Science.

The brain imaging studies were identified based on the following inclusion and exclusion criteria described in Table 1: (1) whole-brain fMRI or MRI studies; (2) fMRI results presented as coordinate-based (x, y, and z) in MNI or Talairach space; (3) studies that used whole-brain analysis to observe the alteration of intrinsic connectivity patterns in pediatric multiple sclerosis; and (4) studies that assessed gray matter abnormalities. The exclusion criteria were as follows: (1) systematic reviews or meta-analyses; (2) behavioral, genetic, or epidemiological studies; (3) single-case MRI or fMRI studies; (4) other imaging techniques (i.e., PET and SPECT) or EEG; and (5) no coordinates. Moreover, all the studies were checked for the MRI criteria for MS diagnosis [17].

All of the included studies were screened to identify additional relevant studies. The narrative, scoping, and systematic reviews and meta-analyses were retrieved and screened to find previous relevant articles in the reference lists. After removing duplicates, the titles and abstracts were assessed to determine if they fulfilled the inclusion and/or exclusion criteria (Table 1), and the full texts of potentially relevant studies were retrieved. Then, we read the full texts to confirm their eligibility. The authors independently conducted a literature search, screening, and methodological evaluation. A consensus about the inclusion or exclusion of a study was reached by brainstorming among the authors.

To assess the quality of the studies included in the present systematic review, we applied the Newcastle–Ottawa scale (NOS scale) [18]. Information was extracted from each included study following the guidelines mentioned above. In particular, we extracted the characteristics of the participants, including the exclusion and inclusion criteria.

To assess the presence of homogeneity in the fMRI results, an activation likelihood estimation (ALE) meta-analysis was performed using GingerAle 3.02 [19] (https://www.brainmap.org/ale/; accessed on 22 April 2024) on the clusters reported in the included studies.

Ginger Ale generates modeled brain activation maps that combine the probabilities of all foci for each voxel, and the resulting maps are combined to obtain a voxel-wise ALE score. Thus, the ALE scores are the convergence of the study results in brain functional anatomical localization. The obtained scores are then compared with an empirical null distribution representing a causal association across the studies [20]. When necessary, all the retrieved coordinates were converted into the stereotaxic space of the Montreal Neurological Institute (MNI).

We used Mango 4.1 [21] (http://ric.uthscsa.edu/mango/mango.html; accessed on 22 April 2024) to visualize the results using a structural MRI T1W MNI template with a 2 × 2 × 2 mm resolution (https://www.brainmap.org/ale/; accessed on 22 April 2024).

Therefore, after the resulting ALE maps were calculated, a series of regions of interest (ROIs), in correspondence with the significant clusters found, were delineated to perform a behavioral analysis, and the behavioral analysis plugin was included in Mango v.2.6 (http://ric.uthscsa.edu/mango/plugin_behavioralanalysis.html; accessed on 22 April 2024). Behavioral analysis is presented for BrainMap’s five behavioral domains (action, cognition, emotion, interoception, and perception) and sixty subdomains. Only z-scores ≥ 3.0 are considered significant (*p* ≤ 0.05, Bonferroni corrected) [22]. This analysis was carried out to identify the domains affected by MS in children.

## 3. Results

### 3.1. Characteristics of the Included Studies

After the selection process, twenty-one studies were included in the present systematic review, as reported in the flowchart depicted in Figure 1 and Table 2 [23,24,25,26,27,28,29,30,31,32,33,34,35,36,37,38,39,40,41,42,43]. Table 3 shows the characteristics of the included studies. All the studies were published between 2009 and 2024, and they were carried out in Europe (n = 15; 71.4%) and Canada (n = 5; 23.8%), with only one carried out in the USA (4.7%). A total of 1237 (817 girls) pediatric subjects participated in the selected studies, with 917 patients with MS and 320 healthy age-matched controls, as reported in the methods of each published article. Among the patients, 595 were girls (64.8%). The mean age of the patients ranged between 11.1 and 16.8 years. Similarly, the mean age of the healthy controls was between 12.2 and 15.5 years. The global sample can be considered principally composed of adolescents.

Most of the studies (n = 13; 61.9%) investigated brain structural changes following MS in children. Eight (38%) were fMRI case–control and case–report studies. Only two (12.5%) included studies were diffusion tensor imaging (DTI) studies.

All the studies reported neurological assessments performed using the expanded disability status scale (EDSS), and all the patients were classified as relapsing–remitting (RR).

### 3.2. Neuropsychological Results

Among the included studies, eleven (52.3%) reported neuropsychological assessments of MS patients. Most of the studies applied the brief neuropsychological battery for children [44], which was designed to study the presence of cognitive impairment in children and adolescents affected by multiple sclerosis. However, despite these findings, the studies also investigated the domains and subdomains Appendix A). The studies focused their attention on the assessment of working memory, sustained attention, abstract reasoning, and language. Three studies reported that it was possible to identify patients with cognitive impairments [26,29,31]. De Meo et al. [26] reported that among patients classified as cognitively impaired, none presented specific alterations in attention. Similarly, Fuentes et al. [27], Hubacher et al. [36], and Margoni et al. [37] reported no working memory alterations when patients and controls were compared or when patients’ results were compared with normative data. Conversely, Rocca et al. [29] found that the cognitive domains most frequently involved were spatial and verbal memory, language, and attention, confirming the results of other studies [31].

Interestingly, Hubacher et al. [36] reported a case series study in which cognitive training was applied using a computerized training tool, BrainStim [45,46]. This training was based on Baddeley’s working memory model [47], which consists of three different modules that target both verbal and visual–spatial aspects. After the training, two out of five participants showed performance improvement, and the remaining participants showed no detrimental performance.

Among these, Green et al. [40] assessed communication skills, including verbal and nonverbal skills and verbal memory, finding that patients had poorer skills than age-matched controls.

### 3.3. Structural Imaging Results

Table 3 summarizes the principal findings as well as the methods reported in the retrieved articles. Most of the included studies (n = 11; 68.7%) described MRI structural alterations found in MS patients. Similarly, the adult population was excluded or used as a group for comparison in some of the included studies [42]. Data concerning the adult patient group were not taken into consideration in the present review.

Studies investigating functional brain imaging alterations were also screened to find structural MRI data. The principal interest observed in the included structural studies was to assess the presence, frequency, and topography of gray and white matter (GM and WM) lesions. Despite the different topographies and frequencies observed in each study, all of them agreed that patients presented a significantly lower whole-brain volume, increased ventricular volume, and a greater number of lesions in both the GM and WM than in healthy controls. GM alterations were investigated by assessing the hypointensities in T1 images, whereas WM alterations were studied using the hyperintensities in T2 images. Interestingly, Margoni et al. [37] reported that the number of lesions was greater in images obtained with phase-sensitive inversion recovery (PSIR) than in those obtained with double inversion recovery (DIR) sequences from a 3T MR scanner. With an interest in WM, several studies have applied DTI and reported a reduction in fractional anisotropy (FA) and an increase in mean diffusivity (MD). More specifically, alterations were observed in correspondence with the median line, involving the precuneus, posterior cingulate cortex, corpus callosum, bilateral superior longitudinal fasciculus, and optic pathway [23,30]. More specifically, structures such as the cerebellum, hippocampus, caudate nucleus, and thalamus have been found to be altered in MS patients.

Indeed, according to Margoni et al. [37], 93% of patients presented with GM lesions in the cerebellum, with a greater frequency in the posterior cerebellar lobe. Similarly, Weier et al. [38] reported that the volume of the cerebellum was significantly reduced in the pediatric MS group. In particular, the cerebellar volume, together with the infratentorial lesion volume, accounted for the extra variance in the WASI vocabulary and the SDTM (Symbol Digit Modality Test) [38]. Lesions in the cerebellum are more common in pediatric MS patients than in adult MS patients [42]. Cerebellar lesions, as well as cervical cord and optic nerve lesions, can also be considered relevant predictors of the relapse rate at baseline [35]. The presence of alterations in the hippocampus was investigated in two different studies. Fuentes et al. [27] were interested in finding cerebral correlates of memory impairment observed in MS. They observed specific alterations in the amygdala and thalamus but not in the hippocampus. However, the hippocampus volume was found to be correlated with word-listening learning. As shown by Rocca et al. [32], MS patients exhibit atrophy in the bilateral cornu ammonis, subiculum, and dentate gyrus and hypertrophy of subcomponents of the dentate gyrus. However, regional alterations in the hippocampus are associated with the level of attention and language, as assessed by neuropsychological tests. These results partially confirm those previously obtained by Fuentes et al. [27].

Kerbrat and colleagues [39] reported a reduction in the global head volume and a reduced thalamic volume following the pediatric onset of MS. However, by combining several MRI techniques and analyzing the thalamus using a laminar approach, De Meo et al. [43] reported that MS patients tended toward a reduction in the thalamus compared with healthy controls. Moreover, they reported that 12 patients presented with thalamic atrophy, with increased levels of fractional anisotropy (FA), T1/T2 ratios, and mean diffusivity (MD) in regions close to the cerebrospinal fluid.

Despite these findings, some studies have investigated cerebral alterations in the course of the disease with one or more follow-ups. Ceccarelli et al. [33], comparing CIS and MS patients, found that the intensity of T2 images of the caudate nucleus remains stable after one year. After two years, in patients receiving steroid therapy, a reduction in WM and total brain volume and an increase in ventricular volume were observed [34].

A comparison between cognitively impaired and cognitively preserved patients revealed that, compared with cognitively preserved patients, cognitively impaired MS patients have a greater occurrence of T2 lesions [29]. However, the hippocampal volume was not found to be affected by cognitive impairment [32].

### 3.4. Functional MRI Studies and ALE Meta-Analysis Results

Among the selected studies, eight (n = 8; 38%) investigated brain functional alterations related to MS in children and adolescents. Four (n = 4; 19%) of these used a resting state, while two (9.5%) used an attentional and a working memory task, and the other two (9.5%) used a finger-tapping motor task. However, one (4.76%) of the studies was a case series study without reporting the resulting coordinates and describing the results case by case [36].

Despite the presence of only a few fMRI-published studies, they attempt to disentangle the principal features observed in pediatric MS. A detailed description of the results and methods, as appeared in the published studies, is shown in Table 3.

**Figure 2:**
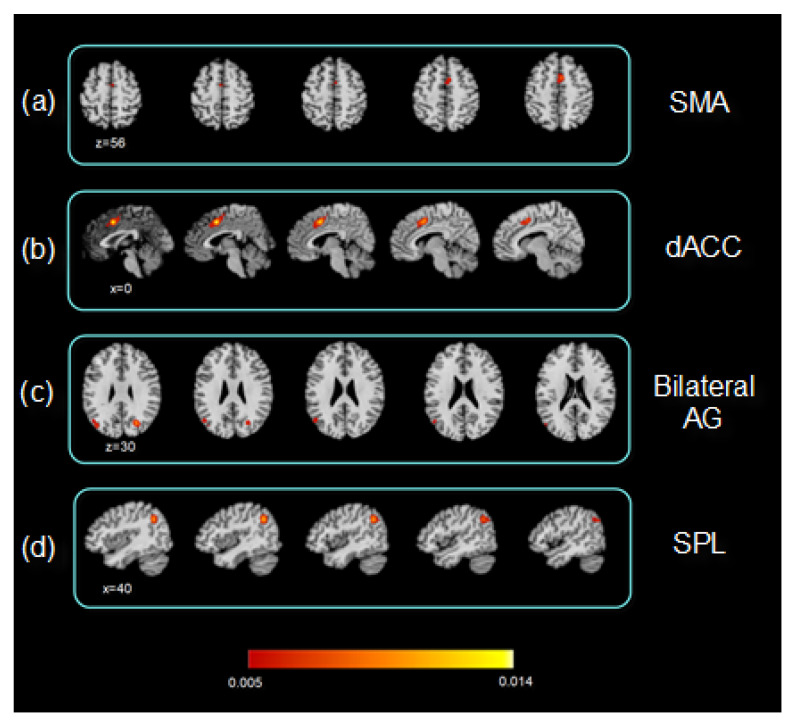
ALE fMRI meta-analysis map results of the included pediatric MS studies. ALE map of the resulting clusters for fMRI studies on pediatric multiple sclerosis. Maps are overimposed on a 2 × 2 × 2 mm MNI template according to neurological conventions. (**a**) The map denotes the cluster corresponding to the left supplementary motor area (SMA); the map in (**b**) includes the cluster corresponding to the dorsal anterior cingulate cortex (dACC); the map depicted in (**c**) denotes the clusters corresponding to the left and right angular gyri; and (**d**) shows the cluster corresponding to the right superior parietal lobe (SPL). The colored bar denotes the corresponding ALE value ranges indicated on the maps (*p* < 0.0005).

Despite the limited number of studies and the heterogeneity in the tasks and analytical techniques used, the extracted coordinates allowed the ALE map calculation (*p* < 0.05, cluster-corrected FWE). The resulting ALE map reveals homogeneous clusters corresponding to the dorsal anterior cingulate cortex (dACC) extending to the supplementary motor cortex (*p* < 0.01). Moreover, bilateral clusters involving the angular gyri and left superior parietal lobe were homogeneous (*p* < 0.0001) (Table 4; Figure 2). After that, a series of regions of interest (ROIs) were delineated in correspondence with the principal homogeneous foci, as depicted in Figure 2. For each ROI, a behavioral analysis was carried out using Mango software. Among the six domains included in the analysis (action, cognition, emotion, interoception, and perception), as described by Lancaster et al. [22], significant z (≥3) scores for some of the domains and subdomains were found for the homogeneous clusters resulting from the meta-analysis calculation. The analysis carried out for the dACC cluster reveals significant results for the domains of cognition, perception, and action. For cognition, significant results were observed for language speech (z = 5.48), semantics (z = 5.57), and phonology (z = 3.004). Moreover, the subdomains of attention (z = 8.30), working memory (z = 5.39), and reasoning (z = 3.29) showed significant results. The perception (somesthesis z = 3.31; pain z = 5.24; and vision z = 3.76) and action (execution z = 6.60; inhibition z = 4.97; and execution speech z = 3.92) domains yielded significant results. The ROI located in the right SPL was significantly related to cognition (working memory z = 4.56; attention z = 3.45; and reasoning z = 4.18) and perception (vision–motion z = 3.30). The subdomain of social cognition was significant for the cluster found in the left angular gyrus, with z = 3.94. No significant results were observed for the cluster found in the right angular gyrus.

## 4. Discussion

The present systematic review aimed to investigate and summarize the principal findings concerning structural and functional brain alterations in children and adolescents affected by multiple sclerosis. Although there is significant interest in multiple sclerosis (MS) among adults, this review focuses on only twenty-one published studies. Most of these studies confirm that the disease is rare in the pediatric population and highlight the challenges of recruiting a sufficient and reliable number of young patients for MRI or fMRI studies. As stated by Yan et al. [3], who analyzed previous epidemiological studies on pediatric MS, most of those studies were carried out in Europe and North America (Canada), as confirmed by our review. Compared with those who experienced onset in adulthood, 2–5% of patients experienced onset in childhood or adolescence [4,48]. According to the data published in the included studies, the patients’ ages ranged between 11 and 15 years, with almost all the patients being adolescents [49]. During adolescence, patients show single-focal symptoms that are more similar to those observed in adult patients and different from the multifocal symptoms observed in children [50]. However, the most reported symptoms are sensorial, motor, and brainstem dysfunction. Most of the included studies reported cognitive impairment in participants with MS. Despite this, they focused their attention on the specific domains affected. Indeed, some of their results were based on the administration of an ad hoc neuropsychological battery developed to assess pediatric MS patients (Brief Neuropsychological Battery for Children (BNBC)). This extensive neuropsychological battery includes more than ten scales and subscales that assess intelligence, memory, and working memory; abstract reasoning; and different aspects of language, from phonetics to denomination to semantics and comprehension. However, the results were heterogeneous in terms of the cognitive domains found to be affected. Specific deficits were observed in visuospatial and working memory, language, and attention (Table 4). Unlike adult patients, as shown by the included studies, language and intelligence are affected by MS [51]. These alterations were more frequently observed in cognitively impaired than in cognitively preserved MS patients. However, in a cohort study [52], 35% of MS pediatric patients presented with cognitive impairment, and the cognitive functions most affected by the disease were motor coordination, visual–motor integration, and information-processing speed.

Most of the included MRI studies used the magnetic resonance protocol suggested in the guidelines for MS [53], in which fast spin echo or turbo spin echo and FLAIR sequences are recommended for assessing brain lesions in MS.

Some of the included studies reported specific structural alterations, with a reduced volume in MS patients [38] or a significantly increased number of lesions in the cerebellum [42]. The cerebellum is a complex anatomical structure implicated in several cognitive functions and behavioral and emotional control [54,55,56,57]. Specifically, since its reciprocal connection with frontal, parietal, and temporal regions, the cerebellum has been found to play a relevant role in language, spatial abilities [58], and working memory. According to Tedesco et al. [59], cerebellar lesions do not disrupt specific functions, making them less efficient. However, acquired lesions during childhood can have a different impact than those acquired during adulthood. Despite the anatomical connection and topography of the cerebellum being effective early in life, cerebellar–cortical circuits continue to develop during adolescence in parallel with improvements in higher-order cognitive functions, such as language, abstract reasoning, and actions. In a recent multicenter study [60], during adolescence, all executive functions were present, and complete development was reached in late adolescence, between 18–20 years. Mid-adolescence (10–15 years) represents a critical period in which the development of these functions is crucial for adulthood. The inferior part of the posterior cerebellar lobe fully develops between 11 and 12 years of age, followed by the anterior lobe and superior portion of the posterior lobe [61].

Spatial cognition and, more specifically, goal-directed spatial navigation are some of the functions modulated by the connection between the cerebellum and hippocampus. The hippocampus comprises several subfields with distinctive histological characteristics, neurochemistry, and functions that play different roles in MS [62,63,64]. The hippocampus and its subfields are widely altered in adult MS patients, with an expanded dentate gyrus [65]. However, these alterations were also observed in pediatric patients with MS. According to the results of the study of Fuentes et al. [27], no differences were found between patients and controls, whereas Rocca and colleagues [32] reported atrophy corresponding to the bilateral cornu ammonis, subiculum, and dentate gyrus and hypertrophy of subcomponents of the dentate gyrus in pediatric MS patients. Similarly, the cerebellum and hippocampus show specific trajectories during development from childhood to adulthood [64]. Several studies have shown that hippocampal volume increases in late childhood and early adolescence, with a peak at 17 years for both girls and boys [66]. A decreased hippocampal volume can result in detrimental outcomes for several cognitive functions during adulthood.

The thalamus is one of the cerebral regions most affected by MS. The thalamus includes several nuclei that are involved in cognitive functions, such as selective attention, in which the pulvinar plays a prominent role [67], and the anterior and medial dorsal nuclei, which are involved in memory encoding and retrieval. Given the high presence of myelin in the thalamus, T1 and T2 lesions are easier to detect than cortical lesions [68]. Thalamic lesions are very common among MS patients (up to 97%), affecting different types of motor and cognitive functions [69]. Most of the lesions observed in adult MS patients are subependymal or perivascular. In pediatric MS, a reduction in the volume of the thalamus has been reported, with lesions involving several portions occurring more diffusely than those observed in adult patients.

Despite the structural alterations, fMRI studies allowed for the decomposition of the cerebral underpinnings of functional alterations in pediatric patients with MS. In addition to the studies conducted on adult patients, the included research examined both resting and task-based brain activity. This led to the identification of a wide range of brain regions consistently involved, including the dACC, the right angular gyrus, and the left superior temporal lobe. The dorsal ACC plays a crucial role in the development of human cognition and behavior. Moreover, the dACC is implicated in the modulation of several cognitive functions, such as motor control, motivation, error detection, working memory, and decision-making [70,71]. The involvement of this region in the modulation of these cognitive functions is enabled by the interplay between different functional networks in which different portions of the cingulate cortex play different and relevant roles. The cingulate cortex, more than a specific or unique hub, can be conceived as a crossroad or intersection of different functional networks [72,73]. Among the portions in which the cingulate cortex can be anatomofunctionally partitioned [74], the dACC is frequently coactivated with the bilateral anterior insula, representing the core of the cingulo-opercular (CO) network [75]. CO and frontoparietal networks play relevant roles in executive functions and processes related to their development during childhood and adolescence [76]. During a task, these two networks work in different but complementary ways. The frontoparietal network is involved in the starting and adjustment of task control, whereas CO maintains the set during the task. This joint activity is also evident during the resting state [77], and its organization changes during neurodevelopment.

None of the studies that we included reported specific alterations in the crosstalk between the bilateral insular cortex and the dACC. However, cognitively preserved patients presented increased functional connectivity in the ACC [29]. Moreover, De Meo et al. [26] reported lower FA values in the tracts connecting the left anterior insula to the anterior cingulate cortex in patients with cognitive impairment than in those without cognitive impairment. Therefore, during an attention load task, decreased deactivation was observed in the ACC when the BOLD response of cognitively preserved patients was compared with that of healthy subjects, suggesting that MS during adolescence may disrupt the functional architecture of the CO network, particularly in patients with cognitive impairment. The development of executive functions depends on several factors, such as synaptic pruning and myelination [78,79].

The relevance of its key role has also been confirmed by the results of the behavioral analysis, which highlighted the role of the dACC in cognition and its subdomains, attention, working memory, and reasoning. These latter genes were found to be altered in children and adolescents with MS, as mentioned above. The dorsal ACC is hypothesized to play a central role in decisions about the allocation of cognitive control on the basis of the highest expected value of control [73]. This complex function seems to be affected by pediatric MS, with sequelae of other cognitive functions. In this way, future studies should assess the role of the cingulate cortex in pediatric patients with MS, using complex multimodal MRI and fMRI paradigms, which could improve the integrity of the NAWM between the anterior cingulate cortex and insula. For instance, recent radiomic approaches, particularly those utilizing T2-FLAIR imaging, have demonstrated promising results in characterizing demyelinating lesions in pediatric multiple sclerosis patients, providing new possibilities for differential diagnosis and aiding in the automation of imaging analysis procedures [80].

Interestingly, four studies [29,31,36,41] investigated brain activity in the resting state, analyzing alterations in networks such as the default mode network (DMN). The DMN was first defined as a set of regions whose activity increased during the resting state [81]. This network is composed of three specialized subsystems: the ventromedial prefrontal cortex (vmPFC), the dorsomedial prefrontal cortex (dmPFC), and the PCC, precuneus, and lateral parietal cortex [82]. All these brain regions are involved in self-related cognitive functions, such as rumination, introspection, self-reflective thoughts, and autobiographical memory, in which theory of mind (ToM) plays a key role [83,84]. The theory of mind allows one to infer the mental state of another, with attributions to their knowledge, beliefs, and emotions [85]. Among the three systems mentioned above, the dmPFC strongly overlapped with ToM-related processes. Unfortunately, none of the four fMRI studies reported specific alterations in functional connectivity in the dmPFC but reported alterations in DMN FC in the comparison between patients and controls. Alterations in social cognition and ToM were observed in cross-sectional case–control studies comparing pediatric patients with MS and healthy controls [86,87,88]. In pediatric patients, worse performance in both affective and cognitive measures has been reported [86], and patients with disease onset before 15 y.o. had significantly lower ToM scores [88]. According to a recent meta-analysis, moderate impairments in empathy, overall ToM, cognitive ToM, and the overlapping domains of cognitive empathy/affective ToM were observed in adults with multiple sclerosis. Although these results revealed an impairment in ToM in both adults and pediatric patients, the associations between impairments in social cognition and alterations in the functional connectivity of the DMN have not yet been studied.

Despite the potential role of the ACC in disentangling some aspects of MS, a series of limitations needs to be acknowledged. We have conducted an ALE meta-analysis on a few studies, demonstrating that MS in pediatric patients is a rare syndrome, with great effort in conducting neuroimaging studies. Importantly, the conclusions drawn from the ALE meta-analysis results should be interpreted cautiously due to the variability in protocols, tasks, and sample sizes across the included studies.

## 5. Conclusions

In summary, this comprehensive review provides insights into the complex landscape of brain structural and functional changes in children and adolescents affected by multiple sclerosis (MS). Despite the rarity of MS in the pediatric population, research highlights the necessity of comprehending its impact on young individuals.

This review suggests that the onset of MS during childhood or adolescence presents unique clinical features, with adolescents often displaying symptoms more similar to those observed in adults. Cognitive impairment has emerged as a significant issue, encompassing deficits in various areas such as visuospatial abilities, working memory, language, and attention. Additionally, the involvement of critical regions such as the cerebellum and hippocampus, which are essential for cognitive and spatial functions, underscores the extensive effects of MS on neurodevelopment during these crucial years.

Structural changes, notably in the thalamus, further emphasize the complexity of MS pathology in pediatric patients. Early detection of structural alterations in these regions, as well as the number of lesions, can help assess the progression of the disease and monitor clinical strategies and interventions.

While MRI studies reveal specific anatomical alterations, functional MRI sheds light on the underlying neural mechanisms driving cognitive dysfunction, with the dorsal anterior cingulate cortex emerging as a pivotal region implicated in various cognitive processes.

These findings contribute to our understanding of MS pathophysiology in young individuals and underscore the importance of early detection and intervention to alleviate cognitive impairments and improve long-term outcomes. In the future, sustained research efforts are necessary to elucidate the intricate relationship between MS pathology and neurodevelopment, ultimately guiding tailored therapeutic approaches for the unique needs of pediatric MS patients.

## Figures and Tables

**Table 1:** Inclusion and exclusion criteria for the selected studies.

Inclusion Criteria	Exclusion Criteria
Design	
Experimental studies Case–control studies	Other designs Systematic reviews
Population	
Children Adolescents	Animals Adults
Intervention	
Neuroimaging techniques Emotional task Cognitive task Resting state Structural MRI	EEG Other imaging techniques
Topic	
Brain activation Brain activity Brain connectivity Gray matter alteration	Brain activation, brain activity, brain connectivity, and gray matter alteration in adult MS patients or adult MS patients with pediatric-onset

Abbreviations: MRI = magnetic resonance imaging; EEG = electroencephalography; MS = multiple sclerosis.

**Table 2:** Newcastle–Ottawa quality assessment scale: case–control studies.

Hits	Selection	Comparability	Exposure
Aubert Broche et al., 2011 [23]	****	*	**
Cirillo et al., 2016 [24]	****	*	**
Rocca et al., 2009 [25]	****	**	***
De Meo et al., 2017 [26]	***	**	**
Fuentes et al., 2012 [27]	****	**	***
Rocca et al., 2010 [28]	****	**	***
Rocca et al., 2014 [29]	***	**	*
Rocca et al., 2016 [30]	**	**	**
Rocca et al., 2014 [31]	***	*	**
Rocca et al., 2016 [32]	***	**	**
Ceccarelli et al., 2011 [33]	***	*	**
Bartels et al., 2019 [34]	***	**	***
De Meo et al., 2021 [35]	**	-	*
Hubacher et al., 2015 [36]	**	-	*
Margoni et al., 2020 [37]	**	-	*
Weier et al., 2016 [38]	****	**	**
Kerbrat et al., 2012 [39]	****	**	**
Green et al., 2018 [40]	****	**	***
Cacciaguerra et al., 2024 [41]	***	**	***
Waubant et al., 2009 [42]	**	**	***
De Meo et al., 2021 [43]	***	**	***

Note: A study can be awarded a maximum of four stars (****) for the selection category and three stars (***) for the exposure category. A maximum of two stars (**) can be given for comparability.

**Table 3:** Principal characteristics and results of the included studies.

Source	N/Age	Neurological Assessment	Magnetic Field/MRI Sequence	Analytical Techniques	Tasks	Lesion Volume	Principal Results
Aubert—Broche et al., 2011 [23]	MS RR: N = 30 (24 girls); mean age = 15.4 ± 2.1 years. HCs: N = 29 (23 girls); mean age = 15.5 ± 2 years	EDSS = Median: 1 (0.0–4.5)	MRI; 1.5 T; 3D T1w RF-spoiled gradient-recalled echo sequence; 2D multislice proton density-weighted (PDw)/T2w fast spin echo sequence	Preprocessing, spatial normalization, and Jacobian calculation GLM	--	Median 3303 mm^3^	Significant volume loss in the MS group in the pulvinar, right anterior nuclei, splenium of the corpus callosum, and globus pallidus. Volume expansion significant in the foramen of Monroe and left lateral ventricle. Regression against disease duration showed significantly correlated volume reductions in the left and right globus pallidus and within the optic tract, from the optic chiasm to the lateral geniculate nucleus and anterior part of the optic radiations
Ceccarelli et al., 2011 [24]	10 CIS and 35 RRMS (27 girls and 18 boys); mean age (SD) 14.7 (2.4) years. 14 HCs (7 girls and 7 boys); mean age (SD) 16.4 (4.7) years	EDSS = Median: 1.0 (0.0–6.5)	1.5 T; dual-echo turbo SE TR = 3300 ms, TE = 16/98 ms	T2 intensity in deep gray matter structures	--	Mean (ml) = 5.2 SD = 7	T2 intensity of the head of the left caudate nucleus differed between pediatric MS patients and HCs (*p* = 0.001)
Fuentes et al., 2012 [27]	Pediatric MS: N = 32 (25 girls); age 16.3. HCs: N = 26 (21 girls); age = 16.4	EDSS = Median: 1.0 (0.0–4.0); neuropsychological assessment	1.5T 3D T1-weighted radiofrequency-spoiled gradient-recalled echo sequence with 1.5 mm thick sagittal partitions, TR = 22 ms, TE = 8 ms. 2D multislice proton density-weighted /T2-weighted fast spin echo sequence with an echo train Length = 58, TR: 3500 ms	Segmentation of brain structures. ANOVA	--	Log T2-LV total brain mean = 3.68 SD = 0.54	MS patients > HCs = Lower whole brain volume Lower normalized volumes in the amygdala (total and left side) and thalamus (left, right, and total)
Rocca et al., 2014 [29]	Pediatric RRMS CP patients: N = 35 (21 girls); age = 15.4. Pediatric RRMS CI patients: N = 16 (9 girls); age = 15.2. HCs: N = 16 (9 girls); age = 14.3	EDSS = Median: 1.5 (0–3.5), neuropsychological assessment	3T fMRI:T2*-weighted single-shot EPI; dual-echo turbo SE; 3D T1-weighted FFE; and pulsed-gradient SE EPI with diffusion gradients applied in 35 noncollinear directions	Functional connectivity analysis. Segmentation. Voxel brain morphometry,	RS	Mean (mL) = 4.8 SD = 6.7	CI patients had an increased probability of harboring lesions in the right thalamus, middle and posterior cingulate cortex, and bilateral parieto-occipital white matter. GM atrophy: CI patients had atrophy of the right precuneus and left middle temporal gyrus. MS patients had decreased FC of the posterior regions of the DMN (Right precuneus). CP patients vs. HCs had decreased RS FC of the right angular gyrus. CI patients vs. both HCs and CP patients had decreased RS FC of the right precuneus. CP patients experienced an increased RS FC of the anterior cingulate cortex
Rocca et al., 2014 [31]	MS CP: N = 28 (19 girls); age = 14.9. MS CI: N = 16 (9 girls); age = 15.7. HCs: N = 27; females: 16; age = 15.3	EDSS = Median: 1.5 (0.0–4.0), neuropsychological assessment	3T fMRI: T-2 *W single-shot EPI: TR = 3000 ms, TE = 35 ms. Dual-echo turbo SE. 3D-T1-W FFE. Pulsed-gradient SE EPI, 35 noncollinear direction	Independent component analysis	RS	Mean (mL) = 4.8 SD = 6.7	Compared to HCs, MS patients showed increased FC between the sensorimotor II network and the DMN and between the WMN and the attention network. They also showed a decreased FC between the WMN and the DMN. The decreased FC between WMN and DMN was corrected for GM atrophy. No differences in FC were found between CP and CI MS patients.
Weier et al., 2016 [38]	MS: N = 28 (21 girls); age = 16.3 ± 2.2. HCs: N = 33 (26 girls); age = 15.5 ± 2.7	EDSS Median: 1.25 (0–4); neuropsychological assessment	1.5T 3D T1-W RF-spoiled gradient-recalled echo; 2D proton density-T2-W fast SE	Segmentation of the human cerebellum and its lobules, combining patch-based label fusion and a template library of manually labeled cerebella of the controls	--	Cerebellar lesion (infratentorial): Median 0.1 (0–4.4) Supratentorial lesion: Median: 4.6 (0.3–35.6)	Cerebral volume was reduced in MS patients. Cerebellar volumes did not differ. Correlation between cerebellar volume and neuropsychological results
Hubacher et al., 2015 [36]	5 patients between 12 and 18 yrs. Case series study	EDSS = NA; neuropsychological assessment	3T; MPRAGE; FLAIR; fMRI: EPI—TR/TE = 2000/23 ms,	GLM; FC	N-back	Mean (mL): Case 1 = 4.2; Case 2 = 5.3; Case 3 = 5.9; Case 4 = 0.85; Case 5 = 9.0	FC between the ventral and the dorsal DMN increased in 2 patients. No differences in the other patients
Cirillo et al., 2016 [24]	RRMS: N = 48; (31 girls); age: 14.9. CP MS: N = 39 (22 girls); age: 14.9. CI MS: N = 8 (5 girls); age: 15.8	EDSS: Median: 1.5 (0–4.0) 9-HPT: nine-hole peg test	3T. fMRI: EPI-TR = 3000 ms, TE = 35 ms; dual-echo turbo spin echo TR/ TE = 2599/16.80 ms. MRI: T1-weighted FFE TR = 25 ms, TE = 4.6 ms	Resting state; seed-based FC; seeds: left and right dentate nucleus of the cerebellum	RS	Mean (mL) = 5.3 SD = 6.7	In MS infratentorial, the T2 lesion volume was approximately 10% of the total T2 lesion volume. Compared with controls, MS patients also showed an increased rest FC between the bilateral dentate nucleus and the left pre- and postcentral gyri
Rocca et al., 2016 [30]	MS patients: N = 11 (9 girls); age = 11.1. HCs: N = 13 (11 girls); age = 12.2	EDSS = Median: 1.0 (0–3.0)	1.5 T, dual-echo fast SE TR = 3500 ms, TE = 15/63 ms. 3D T1-W TR = 22 ms, TE = 8 ms, pulsed gradient spin echo single-shot echo planar imaging, 25 noncollinear directions.	FA and MD	--	Mean (mL) = 2.5 SD = 4.7 Mean (mL) = 7.1 SD = 3.6	MS patients had significantly reduced FA and increased MD in the left and right superior longitudinal fasciculus and corpus callosum
Rocca et al., 2016 [32]	MS CP: N = 41 (26 girls); age = 15.1. MS CI N = 12 (6 girls), age = 15.0. HCs: N = 18 (10 girls); age = 14.0	EDSS = Median: 1.5 (0.0–4.0); neuropsychological assessment	3T; brain dual-echo, turbo SE, and brain 3D T1-weighted FFE	Radial mapping analysis	--	Mean (mL) = 6.3 SD = 7.5	Global hippocampal volume was reduced in MS patients compared to HCs. Compared to HCs, MS patients showed atrophy of the cornu ammonis, subiculum, and dentate gyrus subfields and radial hypertrophy of the dentate gyrus subfield. Regional hippocampal volume modifications correlated with brain T2 lesion volume, attention, and language abilities. Compared to CP patients, CI patients had atrophy of the right subiculum and dentate gyrus subfields
De Meo et al., 2017 [26]	MS CP: N = 44 (30 girls); age: 15.2. MS CI: N = 13 (6 girls); age: 15.1. HCs: N = 14 (8 girls); age: 13.6	EDSS: Median: 1.0 (0.0–4.0); neuropsychological assessment	T2*-weighted single-shot echo-planar imaging scan during the task; DT MRI scan, dual-echo turbo spin echo scan, and 3-dimensional T1-weighted FFE	GLM	CCPT	Mean (mL) = 6.0 SD = 7.8	CI vs. CP patients: lower normalized brain volume, lower WM volume, and lower FA values in the tracts connecting the left anterior insula to the anterior cingulate cortex (ACC) and precuneus. CCPT load condition: CP patients > HCs. Increased activation of the left anterior insula and thalamus and decreased deactivation of the ACC and right inferior frontal gyrus. Increased deactivation of the right precuneus and superior parietal lobule. CI > CP patients decreased activation of the right postcentral gyrus and increased deactivation of bilateral precuneus. CP > CI patients with pediatric MS had increased activity in the parietal and occipital lobes and cerebellum. No significant correlations
Bartels et al., 2019 [34]	MS: N = 37 (18 girls); age = 15; multicenter study—2-year follow-up	EDSS = Median: 0(0–2)	3T; 3D T1-weighted (MPRAGE)	NA	--	Total lesion count: median = 4; range = 1–10	MS patients: significant brain volume loss, GM, and WM volume associated with increased ventricular volume at the first clinical presentation. Patients continue to have further brain volume loss at follow-up (2 years).
De Meo et al., 2021 [35]	N = 123 (brain MRI); N = 115 (cervical cord MRI); 89 girls; age: 14.4 yr. 1-year and 2-year follow-ups	EDSS = Median: 1.5 (0.0–6.0)	1.5 T; BRAIN: FLAIR T2-W, and/or CERVICAL CORD: STIR, T2-W	NA	--	Nr of T2 lesions: mean (range): 30.5 (3–180)	Optic neuritis was observed in 74% of patients. No significant differences in demographic, clinical, and MRI features were observed. Significant nr. of lesions at the brain stem and cervical cord (baseline); 1 yr follow-up: no significant nr. of lesions vs. baseline; 2 yr follow-up: significant nr. of new lesions.
Margoni et al., 2020 [37]	MS = 15 (12 girls); age = 14.1 ± 2.3	EDSS = Median: 1.5 (IQR: 1.0–2.25); neuropsychological assessment	3 T; DIR = resolution 1 × 1 × 3 mm, FOV 230 × 200 mm, TR 13,000 ms, TE 10 ms, TI 3400/325 ms, slices n40, time 3.5 min; PSIR: resolution 1 × 1 × 3 mm, FOV 230 × 200 mm, TR 7000 ms, TE 13 ms, TI 400 ms, slices = 40, and time 7 min.	NA	--	DIR WM lesion, mean nr = 1; PSIR WML, mean nr = 1.6; DIR cortical L, mean nr = 1.1; PSIR cortical mean: nr = 2.3	WM and/or GM lesions were found in the cerebellum in patients. PSIR allowed the identification of a significantly higher number of GM lesions than DIR. Patients had no symptoms of cerebellar dysfunction. The number of supratentorial lesions did not correlate with the number of cerebellar lesions
De Meo, 2021 [43]	MS: N = 70 (44 girls); median age = 15.6. HCs: N = 26 (16 girls); median age = 15.7	Patients’ EDSS = Median: 1.5 (Range: 0.0–4.0)	3T; 3D T1-weighted	MRI analysis Cortical surface reconstruction T1/T2 ratio image reconstruction DTI analysis—FA and MD	--	Median T2 LV, mL (IQR) = 2.9 (1.3–6.3) Median T1 LV, mL (IQR) = 1.7 (0.7–3.8) Median T2 thalamic LV (normalized for thalamic volume), ml (IQR) = 0.0 (0.0–0.8)	Compared to healthy controls, patients had significantly increased fractional anisotropy in the whole thalamus and increased mean diffusivity in thalamic white matter with a trend toward a reduced thalamic volume. In patients, significant fractional anisotropy abnormalities were detected in bands nearest to CSF and in those closest to white matter, while significant mean diffusivity and T1/T2-weighted ratio abnormalities were found in thalamic bands closest to CSF
Cacciaguerra, 2024 [41]	MS: N = 76 (46 girls); age: (median) 14.8 (7.0–17.8). HCs: N = 22 (11 girls); age: 13.8 (8.3–17.9)	EDSS = Median: 1.5 (0.0–4.0) Neuropsychological assessment	3T: T2*-weighted single-shot echo planar imaging (EPI)-T1W	FC	Resting state	Mean = 6.9 SD = 8.5 mL	PedMS showed reduced RS FC in all networks compared to controls, especially in the basal ganglia. In younger patients, reduced RS FC in the basal ganglia, language, and sensorimotor networks is associated with poorer cognitive performance. Older patients showed increased RS FC in the basal ganglia, DMN. In both groups, lower RS FC of the caudate nucleus was associated with poorer executive speed
Rocca, 2009 [25]	MS: N = 17 (11 girls); age: 14.6. HCs: N = 9 (6 girls); age 15.6	EDSS = Median: 1.0, range 5 0.0–3.0	1.5T T2*-W EPI; dual-echo turbo spin echo sequence (TSE); MPRAGE	FC	Finger tapping + rest	Mean = 9.4 ± 13.3 mL	MS patients > HCs increased recruitment of the left (L) primary sensorimotor cortex (SMC). They also showed reduced FC between the L primary SMC and the L thalamus, the L insula and the L secondary sensorimotor cortex, the L thalamus and the L insula, and the L thalamus and the L SII
Green, 2018 [40]	MS: N = 32; 78% girls; age: 16.28. HCs: N = 30; 80% girls; age: 16.01	EDSS = Median: 1.0, range 0.0–4.0 Neuropsychological assessment	---	--	--	--	Greater amygdala volume in patients correlated with parent-reported functional communication and social skills. The right amygdala volume was positively associated with visual memory; the left amygdala volume was a stronger predictor of parent-reported social skills
Waubant, 2009 [42]	MS: N = 41 (51.1% girls); age, mean (SD) 11.4 (4.4).	--	1.5T/3T; T2-weighted FLAIR	Mann- Whitney Multivariate logistic regression	--	Number of T2 LV = 21	Pediatric MS patients showed significant lesions in the juxtacortical, cerebellar, brainstem, and periventricular areas
Kerbrat, 2012 [39]	MS: N = 38; (29 girls); age: 15.2. HCs: N = 33 (28 girls); age: 15.6	--	1.5 T; T1-W gradient-recalled echo; 2-dimensional multislice proton density-weighted/T2-W fast spin echo	--	--	Mean = 6.3 ± 8.1 mL	The intracranial volume z-score was significantly lower in MS patients compared with the HC participants. Patients with MS also demonstrated significant decreases in normalized brain volume z scores. After correction for global brain volume, thalamic volumes in the MS population remained lower than those of HCs
Rocca, 2010 [28]	Pediatric MS: N = 17 (11 girls); age: 14.6. HCs: N = 10 (6 girls); age 15.8. Adult patients/Adult HCs	EDSS = 1.0 (0–3.0)	1.5 T; T2*-W single-shot EPI	Dynamic causal modeling	Finger tapping	Mean = 9.4 ± 13.3	The connectivity of the sensorimotor network was similar in control subjects and pediatric MS patients

*Abbreviations*: IQR = interquartile range; FLAIR = fluid attenuated inversion recovery; EDSS = expanded disability status scale; WM = white matter; GM = gray matter; MS = multiple sclerosis; HCs = healthy controls; CI = cognitively impaired; CP = cognitively preserved; RR = relapsing–remitting; DIR = double inversion recovery; PSIR = phase-sensitive inversion recovery; DT I = diffusion tensor imaging; FA = fractional anisotropy; MD = mean diffusivity; MRI = magnetic resonance imaging; fMRI = functional magnetic resonance imaging; RS = resting state; FC = functional connectivity; N = number; SD = standard deviation; DMN = default mode network; GLM = general linear model; ANOVA = analysis of variance; T = Tesla; CCPT = conjunctive continuous performance task; CIS = clinically isolated syndrome; NA = not available; EPI = echo planar imaging; FFE = fast field echo; TR = repetition time; TE = time of echo; SE = spin echo; MPRAGE = magnetization-prepared rapid gradient echo.

**Table 4:** Coordinates, ALE, Z, and *p*-values resulting from the ALE meta-analysis.

Clusters	Hemisphere	BA	x	Y	z	ALE	Z	*p*-Value
Dorsal anterior cingulate cortex	R	32	2	12	46	0.013638	5.568491	0.00000
Supplementary motor cortex	L	6	−4	−2	58	0.005488	2.99942	0.00135
Superior parietal Lobe	R	7	26	−70	34	0.011553	5.012381	0.00000
Angular gyrus	R	39	42	−58	38	0.010642	4.790189	0.00000
Angular gyrus	L	39	−44	−72	34	0.00792	3.794148	0.00007
Angular gyrus inferior part	L	39	−50	−64	26	0.005742	3.196908	0.00069

*Abbreviations*: BA = Brodmann area; ALE = activation likelihood estimation; R = right; L = left.

## Data Availability

The data are available from the corresponding author upon reasonable request.

## Appendix A. Neuropsychological Assessment as Reported in the Included Studies

**Source**	**Tests/Batteries**	**Cognitive Domains**	**Results**
Cirillo et al. 2016 [24]	Neuropsychological Battery for Pediatric Patients Affected by MS.	-	19% of patients were cognitively impaired.
De Meo et al., 2017 [26]	Modified Card Sorting Test; Oral Denomination Test; Phrase Comprehension Test; Symbol Digit Modalities Test; Spatial Recall Test; Spatial Recall Test Delayed; Selective Reminding Test Consistent Long-Term Retrieval; Selective Reminding Test Delayed; Selective Reminding Test Long-Term Storage; Trail Making Test A/B.	Abstract and conceptual Reasoning Memory Language Attention	23% of MS patients were classified as cognitively impaired. 36% of the cognitively impaired patients and none of the cognitively preserved patients had impairment on attention tests.
Fuentes et al., 2012 [27]	The Test of Memory and Learning—Second Edition (TOMAL-2) Subscales: Word Selective Reminding, Memory for Stories, Abstract Visual Memory, and Facial Memory.	Memory	Overall, lower memory scores in the MS group compared to the HC. All of the patients performed at least within the age-expected level on a measure of delayed, unaided recall of a word list, suggesting that verbal information was well-consolidated for later retrieval.
Hubacher et al., 2015 [36]	Corsi Blocks Backward; Digit Span Backward; Symbol Digit Modalities Test; Test Battery for Attention Performance (TAP).	Working memory functions and basic attention	No working memory deficits compared with normative data.
Rocca et al., 2014 [29]	Brief Neuropsychological Battery for Children (BNBC). Selective Reminding Test (SRT, SRT-Delayed); Spatial Recall Test (SPART, SPART-Delayed); Symbol Digit Modalities Test; Trail Making Test (TMT-A/B); Modified Card Sorting Test; Semantic and Phonemic Verbal Fluency Test; Oral Denomination Test; Token Test, the Indication of Pictures; Battery for the Analysis of Aphasic Deficits.	Memory Language, sustained attention Abstract reasoning	45% of patients were classified as cognitively impaired. The cognitive domains most frequently involved were spatial and verbal memory, language, and attention.
Rocca et al., 2014 [31]	Brief Neuropsychological Battery for Children (BNBC).	Global cognitive functioning, verbal learning and delayed recall, visuospatial learning and delayed recall, sustained attention and concentration, abstract reasoning, expressive language, and receptive language	25 pediatric MS patients were CP.
Margoni et al., 2020 [37]	Trail Making Test—Part B (TMT-B); Symbol Digit Modalities Test—oral version (SDMT).	Executive functioning, cognitive flexibility	Normal performance on neuropsychological tests.
Weier et al., 2016 [38]	Wechsler Abbreviated Scale of Intelligence (WASI FSIQ); Trail Making Test—Part B (TMT-B); Symbol Digit Modalities Test—Oral Version (SDMT); Beery Visual Motor Integration (VMI); Vocabulary Subtest of the WASI; Grooved Pegboard Test.	Intelligence, cognitive flexibility, language, and motor skills	MS patients showed impaired performance (defined as performance falling ≥ 1.5 SD below normative values) in the following subtests: TMT B, VMI, SDMT, and the Pegboard Test.
Cacciaguerra et al., 2024 [41]	Wechsler Intelligence Scale for children (WISC); Wechsler Adult Intelligence Scale (WAIS); Neuropsychological Battery for Children; Symbol Digit Modalities Test (SDMT); Coding Design (CD); Trail Making Test A-B (TMT-A/TMT-B); Semantic Verbal Fluency Test (SVFT); Phonemic Verbal Fluency Test (PVFT).	IQ, executive speed, and expressive language	21.1% of patients failed the CD, 15.8% the TMT-B, 10.5% the TMT-A, 9.2% the SDMT, 3.9% the PVFT, 6.6% the BD, and 1.3% the SVFT.
Green et al., 2018 [40]	Behavior Assessment System for Children, Second Edition: Parent Rating Scale (BASC-2 PRS): Social Skills and Functional Communication Subscales; Wechsler Abbreviated Scale of Intelligence (WASI); Test of Memory and Learning—2nd Edition (TOMAL-2) Subscales: Memory for Stories, Word Selective Reminding, Facial Memory, and Abstract Visual Memory; Children’s Depression Inventory (CDI); Fatigue Severity Scale (FSS); Selective Reminding Test (SRT—Consistent Long-Term Retrieval and SRT—Long-Term Storage); Spatial Recall Test (SPART and SPART-Delayed); Symbol Digit Modalities Test (SDMT); Trail Making Test A-B (TMT-A/TMT-B); Modified Card Sorting Test (MCST); Indication of Pictures Test (IPT); Phrase Comprehension Test (PCT).	Social skills and functional communication Verbal and nonverbal socialization Expressive and receptive communication skills Verbal memory (delayed condition and delayed recall) and visual–spatial memory function	Full-scale IQ was higher in the HC group relative to the MS group, although groups fell within the broad range of average for IQ. Relative to the HC group, patients performed significantly worse on the MFS-D and AVM subtests of the TOMAL-2. They were reported as having poorer functional communication skills on the BASC-2. Performance fell in the impaired range in 9.4% of patients in the MFS-D subtest and only 3.1% of patients in the AVM subtest. Clinically elevated scores were observed in 12.5% of patients regarding functional communication and 3.1% on the Social Skills BASC-2 scale.
Rocca et al., 2016 [32]	Selective Reminding Test (SRT); Spatial Recall Test (SPART)	Long-term storage, consistent long-term retrieval and delayed visuospatial–spatial recall	22.6% of patients were CI. Two patients scored < 70 in IQ, and twelve scored in the inferior range (<90). The cognitive domains most frequently involved were spatial memory, verbal memory, language abilities, attention, and concentration functions.
